# Prolonged delays in leprosy case detection in a leprosy hot spot setting in Eastern Ethiopia

**DOI:** 10.1371/journal.pntd.0010695

**Published:** 2022-09-12

**Authors:** Kedir Urgesa, Naomi D. de Bruijne, Kidist Bobosha, Berhanu Seyoum, Adane Mihret, Biftu Geda, Anne Schoenmakers, Liesbeth Mieras, Robin van Wijk, Christa Kasang, Mirgissa Kaba, Abraham Aseffa

**Affiliations:** 1 Haramaya University, College of Health and Medical Sciences, Harar, Ethiopia; 2 Athena Institute, Faculty of Earth and Life Sciences, Vrije Universiteit Amsterdam (VU University), Amsterdam, Netherlands; 3 NLR, Amsterdam, Netherlands; 4 Armauer Hansen Research Institute, Addis Ababa, Ethiopia; 5 College of Health and Medical Sciences, Department of Nursing, Meda Walebu University, Shashamane, Ethiopia; 6 Erasmus MC, University Medical Center Rotterdam, Rotterdam, The Netherlands; 7 Deutsche Lepra- und Tuberkulosehilfe e.V. (DAHW), Wurzburg, Germany; 8 School of Public Health, Addis Ababa University, Addis Ababa, Ethiopia; Johns Hopkins University, UNITED STATES

## Abstract

**Background:**

Leprosy or Hansen’s disease is known to cause disability and disfigurement. A delay in case detection of leprosy patients can lead to severe outcomes. In Ethiopia, the disability rates caused by leprosy among new cases are relatively high compared to other endemic countries. This suggests the existence of hidden leprosy cases in the community and a delay in timely detection. To reduce disability rates, it is crucial to identify the factors associated with this delay. This study aimed to determine the extent of delay in case detection among leprosy cases in Eastern Ethiopia.

**Methods:**

This cross-sectional explorative study was conducted in January and February 2019 among 100 leprosy patients diagnosed ≤6 months prior to inclusion. A structured questionnaire was used to collect data, including the initial onset of symptoms, and the reasons for delayed diagnosis. Descriptive statistics, including percentages and medians, were used to describe the case detection delay. Logistic regression analysis was carried out to evaluate the predictors of delay in case detection of >12 months.

**Findings:**

The median age of patients was 35 years, with a range of 7 to 72 years. The majority were male (80%) and rural residents (90%). The median delay in case detection was 12 months (interquartile range 10–36 months) among the included patients. The mean delay in case detection was 22 months, with a maximum delay of 96 months. The overall prevalence of disability among the study population was 42% (12% grade I and 30% grade II). Fear of stigma (p = 0.018) and experiencing painless symptoms (p = 0.018) were highly associated with a delay in case detection of >12 months.

**Conclusions:**

Being afraid of stigma and having painless symptoms, which are often misinterpreted as non-alarming at the onset of the disease, were associated with a delay in case detection. This study showed the need to increase knowledge on early symptoms of leprosy among affected communities. Furthermore, it is important to support initiatives that reduce leprosy related stigma and promote health worker training in leprosy control activities.

## Author summary

Leprosy, also called Hansen’s disease, is a chronic infectious disease that may result in devastating disabilities. The disability rate among new leprosy patients in Ethiopia is high compared to other countries. A delay in case detection is a major factor contributing to the development of disability. Late diagnosis also fosters ongoing transmission, increasing the incidence of the disease. We aimed to determine the extent of the detection delay and find the reasons for this delay in eastern Ethiopian districts, endemic for leprosy. One hundred leprosy patients who recently started multi-drug therapy were included in the study. The median detection delay was one year among all patients. About half of the patients had sought treatment from health institutions within one year after the onset of their first symptoms. Longer delays among leprosy patients were associated with a fear of stigma and painless symptoms at an early stage of the disease. This indicates that it is vital to improve community knowledge of leprosy symptoms and to take initiatives that reduce stigma. This should improve health-seeking behaviour and may prevent the development of disability as well as halt transmission.

## Introduction

Leprosy, also called Hansen’s disease, is one of the oldest known infectious diseases [1]. The disease can result in stigmatizing and potentially disabling outcomes [2,3]. *Mycobacterium leprae (M*. *leprae)* is thought to be transmitted from person to person via the respiratory route [1]. In regions endemic for leprosy, 95% of the population do not develop the disease when infected with *M*. *leprae*, assumingly because of a well-functioning immune system [4]. Close and prolonged contact with untreated multibacillary (MB) patients, genetic relationship with the affected person, malnutrition, and low socioeconomic status are associated with disease development [4–6]. The incubation period of leprosy varies from several months to over 20 years after exposure, tempering transmission interruption initiatives [1,7,8]. A delay in the detection of a leprosy diagnosis increases ongoing transmission and allows disease progression, leading to an increased risk of disability [9,10]. Disability caused by leprosy often results in limitations in physical activity, stigmatization and discrimination, leading to decreased social participation [11]. Prolonged delays in diagnosis can consequently affect both the patients and their families [12]. Therefore, the most important factors to improve patient outcomes include early detection of leprosy, adequate treatment and prevention of disabilities, including self-care and the provision of post-exposure chemoprophylaxis to close contacts of patients to prevent development of the disease [13–15].

Ethiopia reached the World Health Organization (WHO) leprosy elimination target of 1 case per 10,000 populations in 1999, leading to the integration of the National Leprosy Eradication Program (NLEP) into routine general health care [16,17]. However, the prevalence and annual incidence of leprosy continues to be relatively high in the past two decades [18]. In both 2018 and 2019, over 3,200 newly diagnosed leprosy patients were reported in Ethiopia, of which 8% in 2018 and 12.8% in 2019 presented with a grade II disability [19–21]. This indicates that the moment of diagnosis and the start of treatment are often delayed [18]. In 2020, 2,591 cases were reported nationally, a decrease which reflects the impact of the coronavirus disease 2019 (COVID-19) pandemic on leprosy services [22–24]. The current passive case detection strategy in the leprosy control program results in high leprosy incidence and fails to detect the hidden cases in the communities [16]. In Ethiopia, misdiagnosis made by insufficiently trained health workers contributes to delayed leprosy detection [18]. Self-treatment, for example by using herbs and visiting traditional healers are found to be associated factors that negatively influence timely detection [12].

It is critical to assess the extent of delay in case detection and to identify barriers that impede patients from presenting signs and symptoms of early leprosy to official health care services. These insights are expected to help setting the agenda in policy making. Therefore, this study aimed to estimate the magnitude of leprosy case detection delay and to identify factors associated with this delay in a leprosy endemic area in Eastern Ethiopia.

## Methods

### Ethics statement and consent to participate

This study was performed in compliance with the international (Helsinki) and Ethiopian research regulations. Ethical clearance was obtained from Haramaya University, College of Health and Medical Sciences Institutional Health Research Ethics Review Committee (IHRERC). It was also approved by the Armauer Hansen Research Institute (AHRI) Ethics Review Committee (ERC). Study participants were given information about the objectives of the study and informed consent was obtained in writing or by thumb print (e.g. in case signing was difficult because of physical disabilities or illiteracy) [25]. When participants aged below 15 years were included, informed consent was obtained from a parent or legal guardian. This is in accordance with IHRERC and AHRI’s ERC. The age of 15 years is also used by the WHO as leprosy indicator definition for children [20–22]. Subjects participated voluntarily and could withdraw from the study at any time without consequences. To minimize stigma, privacy was a priority during the examination of study participants. This was for instance taken into account when choosing the interview location. Participants remained pseudonymous as only a study subject identification number was included during data collection.

### Study area and population

This study was carried out in Fedis district and in Bisidimo Hospital in Babille district, which are both located in the region East Hararghe zone in Oromia Region in Ethiopia. Bisidimo Hospital is a zonal hospital, specialized in leprosy diagnosis and treatment, which serves all populations in East Hararghe. It was established along with the first leprosarium in Harar, Ethiopia. Fedis district was randomly selected from the 12 high-leprosy burden districts in East Hararghe. Fedis district has an estimated population of 133,382 and five health centres that provide general health services including tuberculosis (TB) and leprosy control activities [26,27]. Two health centres, namely Boko Health Centre and Fechatu Health Centre, were selected randomly for this study. According to the zonal health office report, 57 leprosy cases were diagnosed in Fedis district in 2017, which indicates a 4.3/10,000 incidence rate (Zonal TB/Leprosy focal person office communication) [26]. Bisidimo Hospital was selected purposively as it is the only hospital providing dermatology services, including leprosy, in the area.

Leprosy patients of all age and sex groups were eligible for inclusion. The questionnaire was administered to parents or legal guardians in case the included patients were below the age of 15 years. Patients who attended outpatient appointments at Bisidimo Hospital and at selected health centres in Fedis district were also included. Patients who were diagnosed over six months prior to the moment of study inclusion were excluded to prevent recall problems. The study also excluded patients who lived outside the study sites, although treated at Bisidimo Hospital. In addition, leprosy patients needed to give written informed consent to be eligible for inclusion. Using Epi Info 7.2.2 (Centers for Disease Control and Prevention, Georgia, US) − with an expected frequency of disability of 8%, a margin of error of 3%, design, effect 1, at CI 95% − the calculated sample size was 113 [20]. We established disability as a proxy for detection delay to determine the sample size [28].

### Operational definitions

In this study, a leprosy patient is defined as a person with at least one of the cardinal signs of leprosy determined by a trained health professional and who is in need of treatment with multi-drug therapy (MDT) [29]. Leprosy associated disability is categorized according to the WHO leprosy disability grading system. The delay in case detection was defined as the period between the first symptoms of the disease and the diagnosis of leprosy calculated in total number of months [29–34].

Leprosy patients were classified as paucibacillary (PB) or multibacillary according to the Guidelines for Clinical and Programmatic Management of Tuberculosis, Leprosy and TB/HIV in Ethiopia, which links to the WHO definitions [29,35].

“Patient delay” is defined as the period between the moment that a leprosy patient notices the first sign/symptom and the moment the patient visits a first health care provider [34]. A “health system delay” is the duration between the first health care provider consultation and the moment of leprosy diagnosis. In this study, we focus on the total duration of the diagnosis delay, which combines patient delay and health system delay. However, study results were collected from the patient perspective only. For readability reasons, when mentioned “symptoms” in the further text of this article, this should be read as “signs and symptoms”, reflecting both objective and subjective disease manifestations [33].

### Data collection procedure

A structured questionnaire was adopted from our previous work conducted in East Hararghe Zone, Ethiopia [33]. This questionnaire was designed as part of the PEP4LEP project, a research project in which integrated skin screening interventions for leprosy are tested, together with the administration of chemoprophylaxis (single-dose rifampicin post-exposure prophylaxis, SDR-PEP) to contacts of leprosy patients [36]. The questionnaire comprised: (1) patient socio-demographic characteristics; (2) questions to determine the delay in case detection; (3) questions to identify the reasons for delay in case detection; (4) an annex with a picture set to recognize leprosy symptoms and; (5) an annex with a local calendar to translate the patient’s indication of time to a number of months [33]. The questionnaire was prepared in English and translated into the local language (Afan Oromo) and back translated into English by an independent English teacher whose mother tongue was Afan Oromo. The operational suitability (e.g. understandability and length) of the questionnaire for the PEP4LEP study was tested in a pilot in Bisidimo Hospital. Based on these outcomes, adjustments were made to the questionnaire before data collection started. The questionnaire is made available via the online international leprosy knowledge centre Infolep: https://www.leprosy-information.org/resource/case-detection-delay-questionnaire [37].

Data were collected by three leprosy experts. The questionnaire was administered face-to-face. Variables such as socio-demographic characteristics, date of diagnosis, type / clinical form of leprosy, and disability grade before treatment were obtained from the patient’s clinical record.

### Data management and statistical analysis

Data were entered in EpiData 3.1 (Odense, Denmark) and analysed using STATA 13 (Stata Corp LP., College Station, Texas, US). Descriptive statistics such as the mean, median, and percentages were used to describe the socio-demographic characteristics and the extent of delay in case detection. The associations between case detection delay and predictor variables were analysed through univariate analyses. A p-value of <0.25 in the univariate analysis was the criteria for including variables in the multivariable analysis. Further logistic regression analysis was carried out to determine the predictors of delay in case detection. For logistic regression analysis, the outcome variable was defined as a delay of >12 months, at a p-value of <0.05 [38].

## Results

### Socio-demographic characteristics of the study participants

During our data collection period, a total of 140 patients in Bisidimo Hospital and 47 patients in Fedis district were under treatment with MDT. Based on the inclusion and exclusion criteria, 100 leprosy patients were enrolled. The majority of the participants was male (80%) and rural resident (90%), with an illiteracy rate of 77%. The median age of patients was 35 years, with a range of 7 to 72 years. Five percent of the participants were younger than 15 years of age (Table 1).

**Table 1:** Socio-demographic characteristics of participants included in the study, Eastern Ethiopia, from January to February 2019 (number = 100).

Variables	Variable category	Frequency	Percentage (%)
**Study site**	Bisidimo Hospital	61	61
	Health centres	39	39
**Gender**	Male	80	80
	Female	20	20
**Age category (in years)**	<15	5	5
	15–30	41	41
	31–45	30	30
	>45	24	24
**Ethnicity**	Oromo	97	97
	Amhara	3	3
**Religion**	Muslim	97	97
	Christian	3	3
**Residence**	Rural	90	90
	Urban	10	10
**Education level**	Illiterate	77	77
	Primary school	17	17
	Secondary and above	6	6
**Occupation**	Unpaid work at home	11	11
	Farmer	75	75
	Student	11	11
	Civil servant	1	1
	Unemployed	2	2

### Delay in case detection

The median delay in case detection in this study was found to be 12 months, with an interquartile range (IQR) of 10 to 36 months. The mean delay in case detection was 22 months, with a standard deviation (SD) of 19.5 months, with a minimum delay of 2 and a maximum delay of 96 months. About half (47%) of the participants had a delay in case detection of more than 12 months from their first symptom onset. Only 17% of the participants were diagnosed within 6 months after they noticed their first symptom(s) (Table 2).

**Table 2:** Case detection delay and cumulative percentage among study participants, Eastern Ethiopia, January-February 2019.

Delay in case detection (months)	Frequency (number)	Percentage (%)	Cumulative percentage (%)
**< 3**	1	1	1
**3 to 6**	16	16	17
**7 to 12**	36	36	53
**13 to 24**	18	18	71
**25 to 36**	21	21	92
**>36**	8	8	100
**TOTAL**	100	100	100

**Fig 1:**
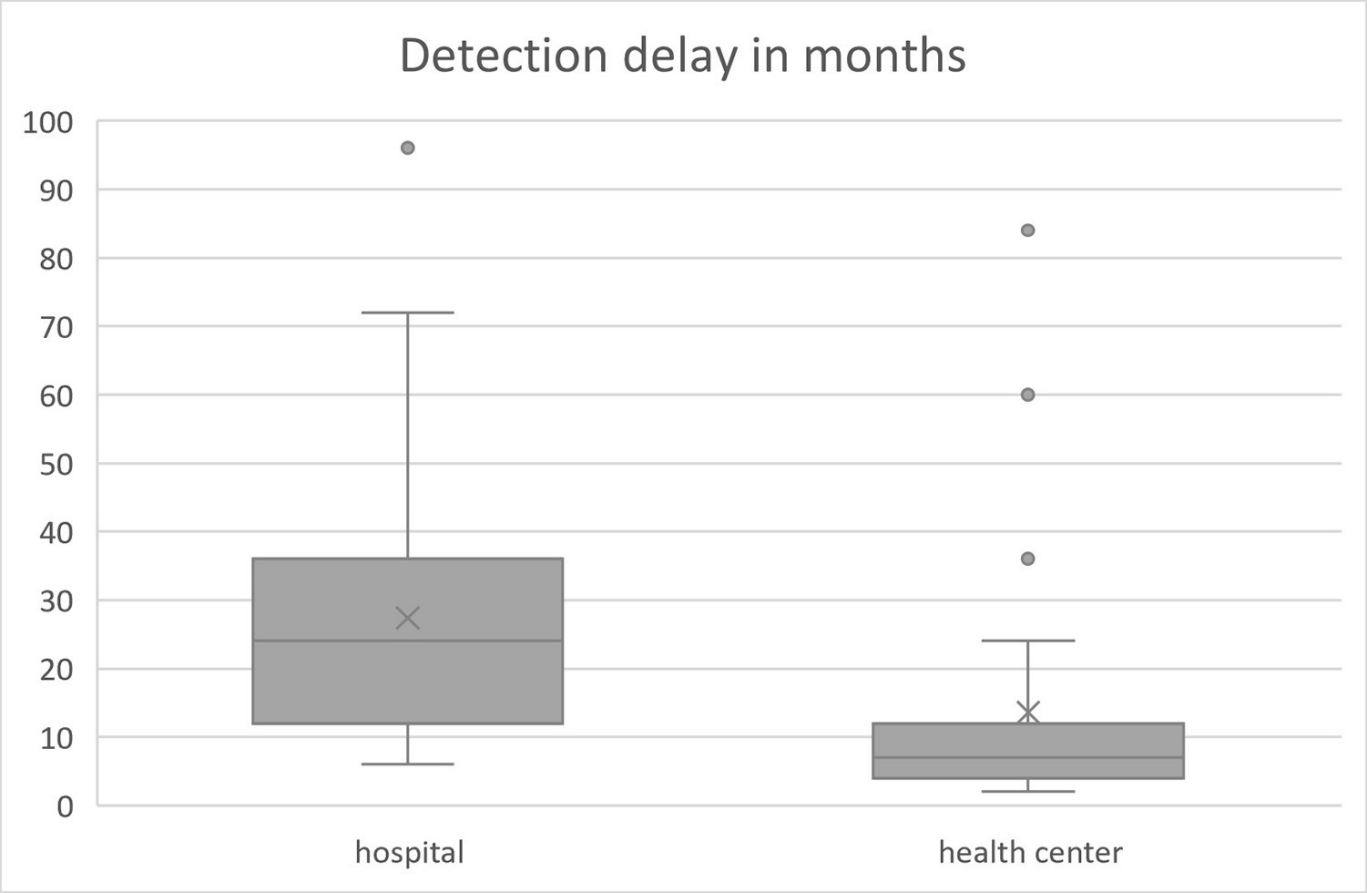
Patient median delays in diagnosis among leprosy patients in East Hararghe Zone, Eastern Ethiopia, January and February 2019.

The proportion of study participants diagnosed in Bisidimo Hospital with prolonged (>12 months) delay in case detection was significantly higher than the number of patients with prolonged delay diagnosed at the health centres (68.9% versus 12.8%, χ2 = 29.98, p<0.000) and patients diagnosed at the hospital had a longer median delay (Fig 1). Most (83.3%) of the patients who presented with a delay in case detection of >12 months had a grade II disability. About half (47.2%) of the study participants with a prolonged delay in presentation were known to have a contact history with persons affected by leprosy in their families. The most frequently noticed symptoms by the study participants were muscle weakness of the hands or feet (91.0%), skin lesion(s) with sensation loss (61.0%), and nerve enlargement (55.0%). The majority (80.9%) of the participants with a prolonged delay explained that this was caused by a fear of stigma.

About a fifth (21.3%) of the participants mentioned painless symptoms of leprosy that did not strike as urgent or severe, as a reason for delay in case detection. In total, 89.4% of the patients were not aware that their experienced symptoms could be leprosy and 55.3% did not seek help earlier because of a lack of money. Over half (65%) of the study participants visited modern health institutions, 59.6% sought treatment from traditional healers and a third (38.3%) visited religious healers after noticing their first symptoms. Only 12.8% of the study participants with a delay in diagnosis of >12 months took no other action between the first appearance of symptoms and the moment of visiting a professional health institution where the leprosy diagnosis was made.

### Factors associated with delays in case detection (>12 months)

In the univariate regression analysis, education level, fear of stigma, shortage of finances, having non-alarming painless symptoms, nerve enlargement, and consulting traditional healers were independent factors associated with a delay in case detection (p<0.25). However, only fear of stigma (AOR = 3.85, 95% CI: 1.26–11.77, p = 0.018) and experiencing non-alarming painless symptoms (AOR = 3.57, 95% CI: 1.24–10.21, p = 0.018) were statistically significant in the multivariable regression analysis (Table 3). Sociodemographic variables such as gender, age, rural or urban residence and religion were not associated with delay in case detection in the univariate regression analysis.

**Table 3:** Association between delays in case detection and related factors among the 100 study participants, Eastern Ethiopia, January-February 2019.

Variables	Category	Delay in case detection		COR [95% CI]	AOR [95% CI]
		<12 months n (%)	≥12 months n (%)		
Education level	Illiterate	45 (84.9)	32 (68.1)		1
	Primary	6 (11.3)	11 (23.4)	2.57 (0.86–7.69)	2.17 (0.58–8.05)
	Secondary and above	2 (3.8)	4 (8.5)	2.81 (0.48–16.29)	2.20 (0.30–16.14)
Visiting traditional healer(s)	No	34 (64.2)	19 (40.4)		1
	Yes	19 (35.9)	28 (59.6)	2.63 (1.17–5.92)*	1.77 (0.64–4.84)
Visiting health institution	No	15 (28.3)	20 (42.5)		1
	Yes	38 (71.7)	27 (57.5)	0.53 (0.23–1.22)	0.51 (0.19–1.39)
Presence of enlarged nerve	No	31 (58.5)	14 (29.8)		1
	Yes	22 (41.5)	33 (70.2)	3.32 (1.44–7.61)*	2.40 (0.84–6.80)
Fear of stigma	No	29 (54.7)	9 (19.2)		1
	Yes	24 (45.3)	38 (80.9)	5.10 (2.06–12.62)*	3.85 (1.26–11.77)**
Lack of money	No	38 (71.7)	21 (44.7)		1
	Yes	15 (28.3)	26 (55.3)	3.14 (1.37–7.19)*	1.52 (0.54–4.27)
Painless symptoms	No	41 (77.4)	25 (53.2)		1
	Yes	12 (22.6)	22 (46.8)	3.00 (1.27–7.11)*	3.57 (1.24–10.21)**

* p<0.25

** p<0.05

Abbreviations: AOR = adjusted odd ratio, CI = confidence interval, COR = crude odd ratio, n = number

## Discussion

In this study, median delay in case detection was found to be 12 months. This finding is similar to the delay described in the study performed by Deps et al. (2006) in Brazil, who also detected a median delay of 12 months [39]. However, in the study conducted by Zhang et al. (2009) in China, a shorter delay of 9.5 months was reported [40]. On the other hand, Nicholls et al. (2003) and Gomez et al. (2018) reported a higher median delay in case detection, of 24 months in Paraguay and 19 months in north-eastern Colombia respectively [32,41]. A study conducted in Ethiopia by Bekri, et al. (1998) revealed that the median delay among leprosy patients in Hararge was 24 months [14].

In this current study, the mean delay in case detection was 22 months. A higher mean delay in case detection was reported by Deps et al. (2006), Engelbrektsson et al. (2019), and Gomez et al. (2018) of 25.3 months in Brazil, 29.8 months in Nepal and 33.5 months in Colombia respectively [39,41,42]. A recent literature review on detection delay by Dharmawan et al. (2021) found a mean delay ranging from 11.5 to 64.1 months [43].

The definition for delayed diagnosis, or prolonged delay in case detection, varies in the literature; six months is referred to as a “long delay”, but some studies advocate for shorter periods of time [32,42,44]. Consequently, Dharmawan et al. (2021) propose a threshold of 6 or 12 months as a uniform definition for detection delay in leprosy [43]. This is in line with the cut-off point of 12 months which was used in this current study.

The known poor performance in diagnosing leprosy by general health workers in Ethiopia may contribute to the detection delay as well as to the observed variation between countries [12,18]. The delay in case detection may also reflect inadequacies in the national leprosy control program [45].

About half of the study participants were diagnosed at a health facility one year after the onset of their first symptoms. A study conducted in another part of Ethiopia by Van Veen et al. (2006) showed that 26% of the patients had a delayed presentation of up to one year [31]. Furthermore, a study conducted by Henry et al. (2016) in Brazil revealed that 84% of the patients sought treatment within 12 months of the onset of their first symptoms [9]. Differences in health service availability, culture and geographical characteristics between these study sites may have contributed to the reported variations in delay [31]. However, the findings of the current study suggest that the detection delay in Ethiopia has decreased over the past decades, when compared to the results found by Amenu et al. (2000) in which 77% of the leprosy patients had experienced a delayed diagnosis of over one year [46]. This difference may be explained by study participants preferring to visit traditional healers previously, leading to a longer delay period, before visiting formal health institutions to seek help [12,46]. This could indicate that the health system has been strengthened over time, and/or that confidence in formal health services has increased among the general population. Another factor which may have contributed is a higher level of community and health workers’ awareness on leprosy [47].

The findings of our study revealed a high prevalence of disability among the study participants; the grade II disability rate was 30%. This is remarkably higher than the prevalence of 8 to 14.8% disability grade II among newly registered national cases from 2018 to 2020, as reported by WHO, and much higher compared to the global average of 5% [20–22]. It is also higher than mentioned in the review made by Deribe et al. (2012), who reported 9.8% grade II disabilities among new patients in Ethiopia [17,48]. Differences may be explained by the fact that the majority (61%) of the current study participants were recruited in a specialized leprosy hospital that usually attracts more severe or more complex patients, or patients who had first sought care elsewhere already, albeit inadequate or insufficient. In our results, patients diagnosed in Bisidimo Hospital had longer delays compared to patients diagnosed in health centres. However, a study conducted in Ethiopia by Shumet et al. (2015), which also took place in a hospital specialized in leprosy care, reported that 24% of the patients included suffered from grade II disability [49]. Studies conducted in other African countries showed a lower proportion of disability grade II among new patients. A study in Nigeria, for instance, reported that 10% of the included leprosy patients had grade II disability; 22% was reported in a study from Malawi [50,51]. When looking at countries from other continents, a lower proportion of grade II disability in newly diagnosed leprosy patients was reported by Gomez et al. (2018) with 15% in Colombia and by Kumar et al. (2015) with 16% in India [41,52]. The magnitude of the disability grade proportion in our study might be attributed to delayed presentation of patients to health services and a late identification of leprosy by health workers. The presence of high disability grade II percentage and long detection delays suggests that many hidden leprosy cases still exist in the study area [53]. In the current study, a considerable number of patients waited a relatively long time before visiting a health institution because of non-alarming painless symptoms. Likewise, the study conducted by Henry et al. (2016) in Brazil reported that about 45% of patients were not diagnosed earlier because they were unaware of the severity of their symptoms [9]. This is comparable to the study conducted in Brazil by Da Silva Souza and Bacha (2003) and in China by Zhang et al. (2009) [40,47]. Both studies showed that unawareness of the disease symptoms was the main determinant of delay in case detection. Doshi et al. (2016) in India also indicated unawareness of symptoms of the disease as a reason for delayed detection [54]. Two other studies performed in India by Muthuvel et al. (2017) and in Nepal by Subedi and Engelbrektsson (2018) similarly reported poor awareness of leprosy symptoms as a reason for delayed presentation [12,34]. The link between symptom misinterpretation and detection delay was further emphasized in the literature review by Dharmawan et al. (2021) [43]. Additionally, Abeje et al. (2016) found that in Ethiopia, the majority for that study surveyed general health workers had poor knowledge of the early signs and symptoms of leprosy, and were unable to perform accurate sensation testing [18]. Over 40% of the health workers in that study also had a negative attitude towards leprosy.

Visiting a traditional healer or performing religious rituals before visiting health institutions was also described in other studies as contributing to case detection delay [12,46]. Therefore, an initiative to raise both community and health workers’ awareness of leprosy seems vital to build knowledge in recognizing the disease in time and to promote a more positive attitude towards leprosy. Among the study participants, 17% had a diagnosis delay of six months or less. It would be interesting to further investigate if more awareness amongst these patients played a role.

Our study also showed that a fear of stigma is an independent predictor of delay in case detection. A study conducted in Brazil by Henry et al. (2016) and in Paraguay by Nicholls et al. (2003) revealed that feared isolation was shown to hinder patients from seeking early treatment [9,32]. A review by Price (2017) pointed out that the stigma surrounding leprosy contributed to later detection among female patients [55]. However, a study conducted in Nepal by Engelbrektsson and Subedi (2018) did not find a significant direct relationship between leprosy related stigma and a delay in case detection [56]. The latter may be explained by differences in the cultural context in Nepal compared to Ethiopia. Culturally sensitive community communication programs on leprosy, which take existing contextual challenges (e.g. illiteracy, preference for traditional medicine, high stigma level) into account are needed in Ethiopia [43].

### Limitation of the study

The participants’ problems in recalling the first leprosy symptoms during the interviews is a challenge [57–59]. However, symptom recall tends to be better than the recall of health-related quality of life or pain intensity. To aid in the recall problems, the national Ethiopian calendar was used, which runs 7 to 8 years behind the Gregorian calendar, counting 12 months of 30 days plus a thirteenth month of 5 to 6 days. In addition, local events like harvest season and large political events were added to the calendar, aiming to potentially minimize problems of recall [60,61]. The included picture set further helped participants in remembering and recognizing signs/symptoms [33]. Medical terms for symptoms are often complex and can differ from words used by patients, which may result in difficulties when recalling these when administering medical questionnaires in research settings [54]. The picture set helped to avoid the use of medical jargon [58]. As advised by de Bruijne et al. (2022), annexes (calendar, photos) should be adapted and validated when the CDD questionnaire is used in other geographical regions or cultural contexts [33].

In addition, the current study included recently detected patients (maximum six months after diagnosis) to minimize the risk on recall problems, because there is an unfavourable relation between the length of the recall period and the recall accuracy (59). In the literature, recall periods used vary from two weeks to over one year and consensus on a reliable period for patients to recall medical events seems to be lacking [53,54].

In the current study, only 20% of the participants were female. Nationally, the percentage of females amongst new leprosy patients has been reported to be 40 [21,22]. Our relatively small sample size may have partly contributed to this. In addition, patients included in this study were patients who self-reported leprosy symptoms and sought medical care. When including participants via active case-finding activities in communities (e.g. skin camps, house visits), the gender balance may be different. Peters and Eshiet (2002) described a hospital-based study in Nigeria, in which female participants (33%) experience a detection delay of almost twice as long as their male counterparts [62]. The relative low number of females included in our study may therefore have impacted our results regarding the detection delay. Dijkstra et al. (2017) performed a literature review on existing gender inequalities in the social impact of leprosy and described stigma as a major cause [63]. Other reasons for underreporting in women listed by Price (2017) included limited mobility, lower educational levels, economic dependence and low social status of women [55]. It is therefore important to focus on gender sensitivity in leprosy interventions, paying extra attention to accessible health services, (community) health education and stigma reduction.

Furthermore, it is difficult to generalize the results of this study because of the small sample size. This may also be reflected in the 5% of patients below the age of 15 years which were included. On a national level, this is around 15% [21,22]. Considering the low number of new leprosy patients per health centre, an extended field study is advised to come to a better case detection delay estimate and to get a better understanding of the reasons for the delay. It would also be interesting to investigate the causes of health system delay more in-depth for the regions included in this study [34].

## Conclusions

Our study showed a prolonged case detection delay of over 12 months in half of the study population of leprosy patients in Fedis district and from Bisidimo Hospital in Ethiopia. Fear of stigma and painless symptoms, which were misinterpreted as non-alarming, were predictors of a delay in case detection. Among the patients who presented with a delay of over 12 months, 83% had a grade II disability. Therefore, trained primary level health workers should focus on context-specific interventions that improve the health-seeking behaviour of the community. Providing health education to communities is vital to support early recognition of leprosy symptoms and to reduce discrimination and stigmatization of leprosy patients and their families. These interventions are expected to cause a decrease in the delay of case detection and reduce the risk of disability and ongoing transmission of the disease.
